# Concentration of Serum Biomarkers of Brain Injury in Neonates With a Low Cord pH With or Without Mild Hypoxic-Ischemic Encephalopathy

**DOI:** 10.3389/fneur.2022.934755

**Published:** 2022-07-07

**Authors:** Pratima Gaulee, Zhihui Yang, Livia Sura, Haiyan Xu, Candace Rossignol, Michael D. Weiss, Nikolay Bliznyuk

**Affiliations:** ^1^Department of Pediatrics, University of Florida, Gainesville, FL, United States; ^2^Department of Emergency Medicine, University of Florida, Gainesville, FL, United States; ^3^Department of Agricultural and Biological Engineering, Biostatistics and Statistics, University of Florida, Gainesville, FL, United States

**Keywords:** HIE, UCH-L1, GFAP, NFL, miRNAs, Tau

## Abstract

**Objective:**

To determine the concentrations of four neuroprotein biomarkers and 68 miRNAs in neonates with low cord pH and/or mild hypoxic-ischemic encephalopathy (HIE).

**Study Design:**

A prospective cohort study enrolled neonates with low cord pH (*n* = 18), moderate-severe HIE (*n* = 40), and healthy controls (*n* = 38). Groups provided serum samples at 0–6 h of life. The concentrations of biomarkers and miRNAs were compared between cohorts.

**Result:**

The low cord pH and moderate-severe HIE groups had increased concentrations of GFAP, NFL and Tau compared to controls (*P* < 0.05, *P* < 0.001, respectively). NFL concentrations in mild HIE was higher than controls (*P* < 0.05) but less than moderate-severe HIE (*P* < 0.001). Of 68 miRNAs, 36 in low cord pH group and 40 in moderate-severe HIE were upregulated compared to controls (*P* < 0.05). Five miRNAs in low cord pH group (*P* < 0.05) and 3 in moderate-severe HIE were downregulated compared to controls (*P* < 0.05).

**Conclusion:**

A biomarker panel in neonates with low cord pH may help clinicians make real-time decisions.

## Introduction

Mild hypoxic-ischemic encephalopathy (HIE) in neonates is defined as a neonate with a low cord pH and a perinatal adverse event who meets one or more abnormal category-modified Sarnat staging criteria within 6 h of birth and does not have evidence of moderate to severe encephalopathy (1). Classically, neonates with mild HIE have been excluded from neuroprotective intervention studies due to their lack of adverse long-term outcomes based on early data (2). However, recent studies have cast doubt on withholding neuroprotective interventions from neonates with mild HIE. A systematic review involving 20 studies showed abnormal long-term outcomes in at least 25% of neonates with mild HIE (3). In addition, mild HIE has been associated with abnormal MRI findings, specifically involving the watershed areas of the brain in 20–40% of neonates with mild HIE (4). The PRIME study examined neonates with mild HIE and found that 52% had adverse short-term outcomes as defined by an abnormal amplitude EEG, an abnormal brain MRI at <30 days of life, and/or an abnormal neurological exam (5). Neonates involved in the PRIME study were assessed at 18–22 months of age for neurodevelopmental outcomes and 16% were found to have disability. Of those with disability, 40% had Bayley scores <85 in the domains of cognition, motor, or language (6). In the NICHD trial, 25% of neonates with perinatal acidosis who did not qualify for therapeutic hypothermia had abnormal short-term outcomes including feeding difficulties, an abnormal MRI at discharge, seizures, or a need for gastrostomy tube feedings (7). Even a mild degree of acidemia at birth was associated with higher odds of short-term morbidity compared to neonates with no acidemia. The risk of morbidity progressed as umbilical artery pH decreased (8).

Due to the decreased severity of injury in neonates with mild HIE, not all neonates need a neuroprotective intervention such as hypothermia therapy. However, we currently do not have a reliable method of differentiating those at high risk of long-term injury. Additionally, cooling all neonates with mild HIE may lengthen their hospital stay without producing a universal benefit. In a retrospective study done in Canada, neonates with mild HIE received either hypothermia treatment or standard normothermia. Neonates treated with hypothermia had a longer hospital stay in tertiary care NICUs and required longer durations of respiratory support (invasive and non-invasive) but had lower odds of brain injury measured by brain MRI (9). An effective screening tool is needed to identify neonates who may benefit from neuroprotective interventions among those with mild HIE and severe acidemia, which will avoid unnecessary treatments.

Biomarkers are a tool that might help selectively identify neonates with acidemia and/or mild HIE with brain injury. These neonates could have adverse long-term outcomes and would benefit from early neuroprotective interventions. Brain injury biomarkers are molecules released into the blood after brain injury that can help predict the location, degree, or timing of injury. Extensive research is being conducted on biomarkers as a bedside tool for clinicians working with neonates with moderate and severe HIE to assess severity, timing, pattern of brain injury, and correlations with biomarker serum concentrations and long-term outcomes (10). Glial fibrillary acidic protein (GFAP) is a cytoskeleton intermediate filament specific to astrocytes involved in regeneration and gliosis after brain injury (11). GFAP concentrations are higher in neonates with moderate-severe HIE vs. mild HIE within 0–6 h of birth and is predictive of motor developmental outcomes (12). Ubiquitin carboxy terminal hydrolase (UCH-L1) is a neuronal specific protein predominantly concentrated in cortical neurons, resistant to endogenous brain proteases, and elevated after acute cortical damage (13, 14). Previously, our group found that UCH-L1 serum concentrations were elevated in neonates with HIE compared to controls. UCH-L1 concentrations were elevated within 0–6 h of age and continued to be higher than control concentrations for the first 24 h of sampling. Also, the concentrations of UCH-L1 at 12 h correlated with adverse developmental motor outcomes in neonates with HIE (12). Tau protein is a microtubule-associated protein abundant in neuronal axons that accumulates intracellularly in HIE and leads to axonal injury. The presence of Tau protein in serum correlates to brain injury up to 5 days after insult and is related to functional outcomes at 1 year (15). Neurofilament Light Chain (NFL) is the most abundant cytoskeleton protein in myelinated axons of CNS (16). NFL is released after axonal injury into the CSF and blood. As a biomarker at 24 h, it can predict unfavorable MRI outcomes in neonates with moderate-severe HIE undergoing hypothermia treatment (17).

A miRNA is a non-coding RNA 21-22 nucleotides in length that binds to the 3'-untranslated region of a target gene and regulates gene expression by inducing mRNA degradation or by inhibiting translation (18, 19). Concentrations of miRNAs are altered in neonates with hypoxia-ischemia (20). MiR-210 is one of the major miRNAs involved in the hypoxic response by inducing microglial activation and regulating microglia-mediated neuroinflammation in neonates with HIE (21). Because miRNA and neuroprotein biomarkers are found in neonates with moderate to severe HIE, a biomarker panel may provide clinicians with objective information that will help them identify neonates with mild HIE at risk for brain injury.

As a first step in moving a panel of neuroprotein biomarkers to the bedside to allow the clinician to selectively identify neonates with mild HIE who would benefit from neuroprotective interventions, we evaluated the serum concentrations of GFAP, UCH-L1, NFL, and Tau proteins in neonates. The neonates who participated in our study had low cord pH and did or did not have evidence of mild HIE on neurologic exam and were compared with healthy controls and neonates with moderate-severe HIE. We also screened a panel of 68 miRNAs that could complement the neuroprotein biomarkers. We compared the concentrations of these miRNAs from healthy controls with neonates with low cord pH group and moderate-severe HIE. We hypothesized that the neonates with low cord pH, with or without evidence of mild HIE, would have lower serum concentrations of these biomarkers than neonates with moderate-severe HIE. The serum concentrations of any neonate with HIE would be higher than the serum concentrations of healthy controls. Furthermore, we compared the biomarker panel concentrations with physiologic parameters and neurologic exams. We hypothesized that serum concentration of miRNAs would be different in the healthy control neonates compared with the neonates with low cord pH or moderate-severe HIE.

## Materials and Methods

### Patient Population

The University of Florida Institutional Review Board and Ethics committee approved all aspects of this study.

#### Low Umbilical Cord pH With or Without Evidence of Mild HIE

In the Neonatal Intensive Care Unit (NICU) at UF Health Gainesville, a sample of blood was obtained from the umbilical artery and vein of all inborn neonates. The worse pH was taken into consideration as there can be errors during labeling or identifying vessels. If the neonate's umbilical cord pH was between 7.11–7.15, a Neonatology Fellow or Nurse Practitioner was notified, and bedside serial neurologic exams using the modified Sarnat scoring was performed every 1–2 h up to 6 h of life to assess for changes in the Sarnat score. If neonates had a worsening Sarnat score, neonates were transitioned to the NICU to be evaluated for therapeutic hypothermia. If the cord pH was ≤ 7.1, neonates were transitioned to the NICU for closer monitoring, collection of a blood sample for analysis (CK, CK-MB, troponin, PT/PTT, fibrinogen, LFT, ABG, lactate), and aEEG monitoring. Regardless of the pH, if the neonate had a normal neurologic exam, normal labs and/or an aEEG with no evidence of hypoxic-ischemic injury for the 6-h monitoring period, the neonate was transitioned back to the mother. Neonates that evolved and met the criteria for therapeutic hypothermia during the 6-h observation period received cooling therapy (see below). Neonates with a pH between 7.11–7.15 had clinical labs drawn at the discretion of the attending physician. While obtaining the clinical samples, an additional 1 ml of arterial blood in BD vacutainer gold top serum separator was obtained for serum neuroprotein biomarker evaluation. The study team had 24 h from the time of sample collection to obtain informed consent for the biomarker study. If the mother did not consent, the sample was discarded. Neonates who had 1–2 abnormal findings on the modified Sarnat exam met the criteria for mild HIE.

#### Moderate to Severe HIE Neonates

Neonates with HIE who were eligible for hypothermia therapy were enrolled in our biorepository. Entry criteria for hypothermia therapy included a gestational age of 35 weeks or greater, a birth weight of 1.8 kg or greater, and ≤6 h of age. Enrolled neonates had evidence of encephalopathy as defined by seizures or three or more abnormalities on a modified Sarnat exam (6). Evidence of hypoxia-ischemia was defined by (1) a pH of 7.0 or less and/or a base deficit of >16, or (2) a pH between 7.01 and 7.15 and/or a base deficit between 10 and 15.9, or (3) an acute perinatal event (cord prolapse, heart rate decelerations, or uterine rupture) and an APGAR of 5 or less at 5 min of life or mechanical ventilation at 10 min of life if no blood gas was available (6). Neonates who were transitioned to the NICU for a cord pH of ≤7.10, declined over 6 h, and met the criteria for therapeutic hypothermia were also included in this cohort. Prior to initiation of therapeutic hypothermia, neonates had blood drawn to assess CK, CK-MB, troponin, PT/PTT, fibrinogen, liver function, ABG, and lactate at 0–6 h of life. During that period, 1 ml of arterial blood in BD vacutainer gold top serum separator was obtained for biomarker evaluation.

#### Control Neonates

Healthy full-term neonates born at University of Florida Health, Gainesville, FL, with APGAR scores of 8 or more at 5 min of life served as controls. A single sample of blood was collected from the umbilical cord (artery or vein) at the time of birth for the assessment of biomarkers.

### Blood Processing

Serum samples (1 ml) were collected using a 3.5 ml serum separator tube (SST, BD Vacutainer^®^ SST Plus Blood Collection Tube, Franklin Lakes, NJ). Samples were allowed to clot in an upright position at room temperature for 30 min in the processing lab (45 ± 15 min from time of collection), then centrifuged at 1,200 g at room temperature for 15 min in a fixed angle centrifuge rotor. Immediately following centrifugation, the serum was transferred from the SST using a disposable transfer pipette into a 2 ml cryovial with a red cap insert (USA Scientific, Orlando, FL). The serum samples were stored at 4 °C. A fiberboard cryogenic storage box (Fisher Scientific, Pittsburgh, PA) was used to store serum aliquots at −80°C until assay analysis. Blood collection from neonates was completed in accordance with common practice as well as state and federal regulations.

### Biomarker Analysis

#### Enzyme-Linked Immunosorbent Assay (ELISA)

Investigators blinded to the clinical data measured GFAP, UCH-L1, NFL, and Tau concentrations using the same batch of reagents using a Simoa^®^ Neurology 4-plex A Assay kit in an SR-X™ immunoassay analyzer (Quanterix Corp, Boston, MA, USA), which runs ultrasensitive paramagnetic bead-based ELISAs.

### MiRNA

Multiplex miRNAs were measured using a Firefly miRNA particle assay system coupled with a portable flow cytometer/reader (Guava^®^ easyCyte™ 6HT, Millipore, Burlington, MA). Sixty-eight neurological miRNA targets were screened using FirePlex™ miRNA neurology panel V2 (cat# ab218371, Abcam, Waltham, MA). Briefly, according to the protocol from the manufacturer, RNA samples were extracted by incubating 20 ul serum, 20 ul RNase-free water, and 40 ul Lysis Mix for 45 min at 60°C while shaking. Then, in a 96-well filter plate, the Firefly miRNA kit was incubated with 25 ul Hybridization Buffer and 25 ul extracted RNA at 37°C for 60 min. After rinsing to remove unbound RNA, 75 ul of Labeling Buffer was added to each well, and the plate was incubated for 60 min at room temperature. Adapted-modified miRNAs were released from the particles by incubating with Rnase-free water for 30 min at 62.5°C, and PCR amplified using a fluorescently-labeled primer set. The PCR product was hybridized to fresh Firefly particles at 37°C for 30 min and re-suspended in Run Buffer for readout. Particles were scanned on an EMD Millipore Guava 6HT flow cytometer. The raw output was background subtracted, normalized using the geometric mean of the normalizer miRNAs, and log transformed.

### Statistical Analysis

Data analyses were performed using SAS software (9.4, SAS Institute Inc, Cary, NC) and R (R 4.0.3 and 1.3, 2014). Logarithmic concentrations of biomarkers were used to respond to skewness toward large values. In our analysis, one-way ANOVA model was used. Since the data does not always satisfy the normality or homogeneity of variances assumptions of the classical normal-theory inference, non-parametric statistical testing (Kruskal-Wallis) was used to test the null hypothesis of no group difference for each biomarker; this was used as the baseline approach. To confirm the results from non-parametric tests that may be less powerful than parametric tests when model assumptions are met (at least, approximately), we also performed classical parametric ANOVA tests (*F*-tests) with and without the assumption of equal variances. Specifically, for each biomarker homogeneity of variances (among groups) hypothesis was tested using Bartlett's test; if the null hypothesis (of equal variances) is not rejected, classical (unweighted) ANOVA tests may be used; else Welch test would be used. In the retrospect, the outcomes/conclusions of this parametric testing strategy matched closely with non-parametric testing using Kruskal-Wallis test. Analysis of variance (ANOVA) was performed to compare the expression of the 68 differentially expressed miRNAs in mild HIE and controls.

## Results

### Patient Population

The demographics of neonates with a low cord pH, with or without evidence of mild HIE and moderate-severe HIE, were comparable in gestational age, birth weight, sex, and Caesarian section rates (Table 1). All neonates in the low cord pH group were inborn, whereas 50% of the neonates in the moderate-severe HIE group were outborn and transferred for therapeutic hypothermia. The APGAR scores in the moderate-severe HIE group at 1 and 5 min were lower, 1.89 ± 1.55 and 4.78 ± 1.8, respectively, compared to neonates in the low cord pH group, 3.8 ± 3.14 and 7.2 ± 1.8, respectively (Table 1, *P* < 0.05).

**Table 1:** Demographic data.

	**Low cord pH** **(*n =* 18)**	**Moderate/severe** **HIE** **(*n =* 40)**	***p*-value**
Gestational age	38.7 ± 1.46	38.22 ± 1.87	0.2
Gender (Male)	12(67%)	27 (67%)	
Birth weight (grams)	3419 ± 516	3367 ± 825	0.77
Outborn	0	35%	
Mode of delivery C section	9 (50%)	20 (50%)	1.0
APGAR at 1 and 5 min	3.8 ± 3.14 7.2 ± 1.8	1.89 ± 1.55 4.78 ± 1.8	0.02 <0.001

Among the 18 neonates in the low cord pH group, 10 neonates had evidence of a low cord pH without evidence of encephalopathy. Eight neonates in the low cord pH group had evidence of mild HIE, Sarnat 1. We further compared the demographics of the low cord pH neonates without HIE with neonates with mild HIE and did not find a difference in gestational age, birthweight, or APGAR scores (Table 2). The mean cord pH for the low cord pH group without evidence of HIE was 7.05 ± 0.55 compared with 7.07 ± 0.05 in the low pH group with mild HIE. The mean cord base deficit for the low cord pH group without evidence of HIE was −11.25 ± 3.5 vs. −11.41 ± 3.5 in neonates with a low cord pH and mild HIE. Nine of the ten neonates in the low cord pH group without evidence of mild HIE had evidence of respiratory acidosis (defined by PaCo2 > 60) in cord blood whereas 7 out of 8 neonates in the low pH group with mild HIE had evidence of respiratory acidosis with a mean PaCo2 of 84.2 ± 11.3 and 74.66 ± 16.5, respectively.

**Table 2:** Demographic data.

	**Low Cord pH** **without HIE** **(*n =* 10)**	**Low cord pH** **with mild HIE** **(*n =* 8)**	***p*-value**
Gestational age	38.4 ± 0.96	39.12 ± 1.9	0.36
Birth weight	3338 ± 543	3521 ± 495	0.46
Cord pH	7.05 ± 0.55	7.07 ± 0.05	0.33
Cord base deficit	−11.25 ± 3.5	−11.41 ± 3.5	0.92
Cord PaCo2	84.2 ± 11.3	74.66 ± 16.5	0.18
APGARS at 1 min	4.3 ± 0.6	3.25 ± 2.4	0.49
At 5 min	7.3± 2.11	7.12 ± 1.45	0.83

### Serum Concentration of Biomarkers in Neonates With a Low Cord pH Compared to Control Neonates and Neonates With Moderate to Severe HIE

**Figure 1:**
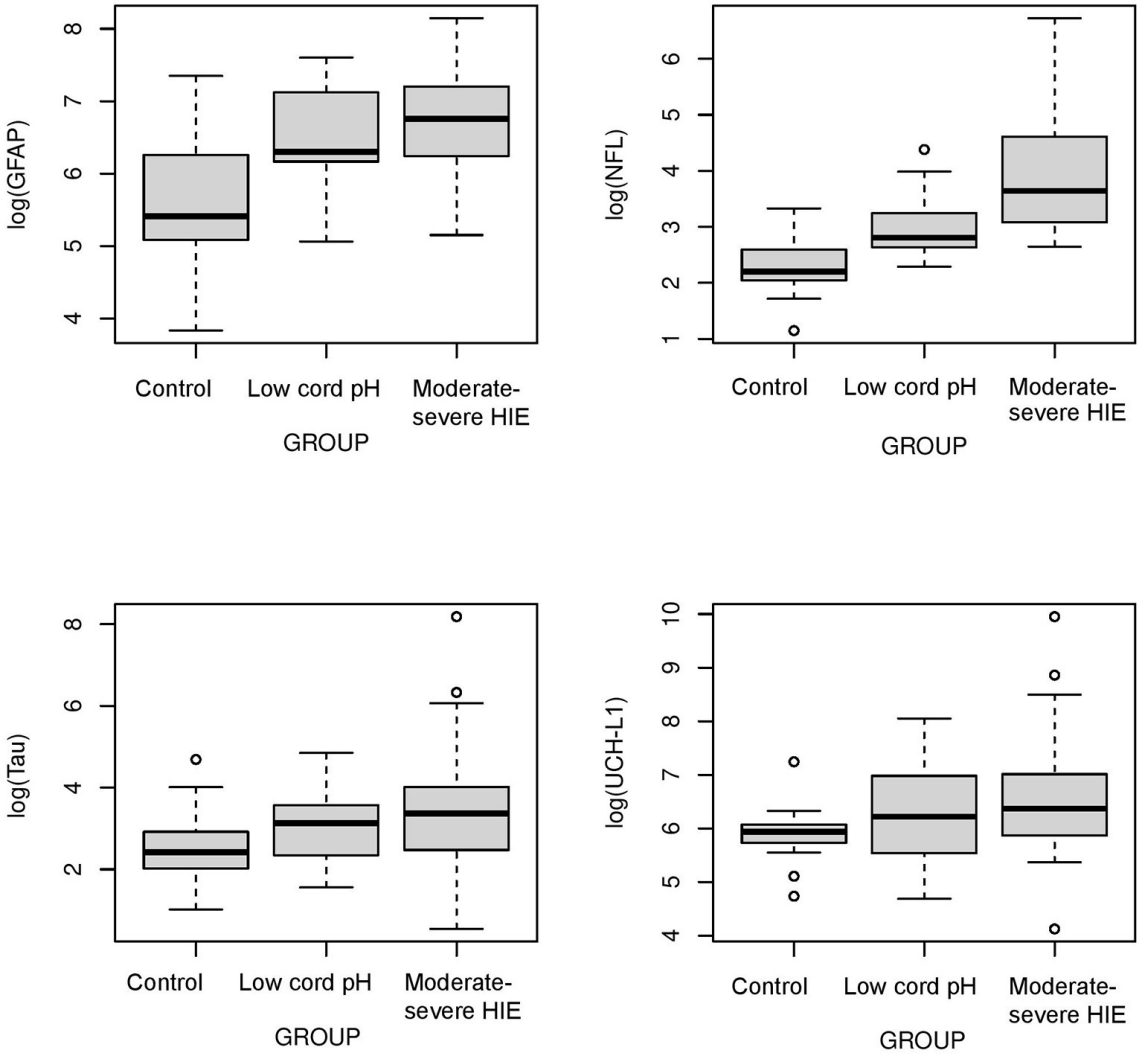
*Comparison of neuroprotein Biomarkers GFAP, NFL, Tau, UCHL-1 among Control, low cord pH neonates and moderate-severe HIE neonates*. Compared to Control group, serum concentrations of GFAP, NFL and Tau were higher in low cord pH group (*P* < 0.05) and moderate -severe HIE group (*P* < 0.001). The concentrations of UCHL-1 were increased in neonates with moderate-severe HIE compared to control neonates (*P* < 0.001). Concentrations of UCH-L1 was not different between controls and neonates with low cord pH, with/without mild HIE, at 0–6 h of life. Compared to moderate-severe HIE, serum concentration of NFL was lower in mild HIE group (*P* < 0.01).

**Figure 2:**
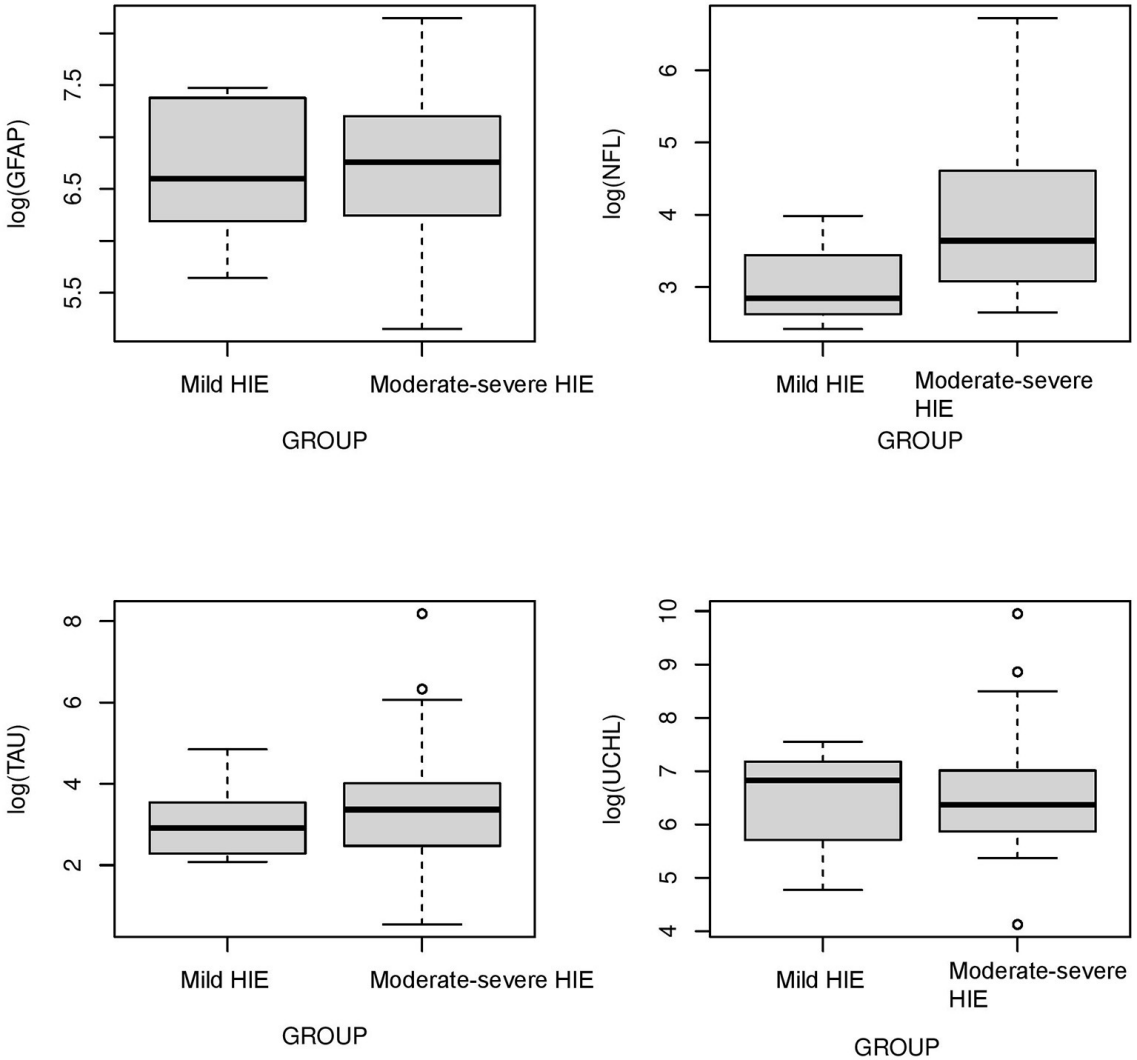
*Comparison of neuroprotein biomarkers GFAP*, NFL, Tau, and UCHL1 *between mild HIE and moderate-severe HIE*. Compared to moderate-severe HIE, serum concentration of NFL was lower in mild HIE group (*P* < 0.01). Concentration of GFAP, Tau and UCHL-1 was not different between neonates with mild HIE and moderate -severe HIE.

The concentrations of biomarkers were measured from each cohort using serum samples obtained at 0–6 h of life. The serum concentrations of GFAP, NFL and Tau were increased in neonates with low cord pH compared to control neonates (*P* < 0.001, Figure 1). Serum concentration of NFL was lower in neonates with a low cord pH compared to neonates with moderate-severe HIE (*P* < 0.01, Figure 1) but GFAP, tau and UCHL-1 concentration were similar between neonates with low cord pH and moderate-severe HIE. Further comparison of neonates with mild HIE (*n* = 8) vs. neonates with moderate-severe HIE showed that NFL was also lower in neonates with mild HIE compared to neonates with moderate-severe HIE (*P* < 0.01, Figure 2). GFAP, NFL, UCH-L1 and Tau concentrations were increased in neonates with moderate to severe HIE compared to control neonates (*P* < 0.001, Figure 1). The mean concentration of four biomarkers in 3 cohorts of control neonates, low cord pH group and moderate-severe HIE is outlined in Table 3.

**Table 3:** Comparison of Mean values of biomarker concentration among 3 cohorts.

**Cohort**	**Biomarkers**			
	**GFAP**	**NFL**	**Tau**	**UCH-L1**
Control group	342.91 ± 307.8	11.34 ± 5.9	16.84 ± 18.9	400.71 ± 196.5
Low cord pH neonates	850.6 ± 572.1	23.9 ± 18.5	27.9 ± 28.1	802.2 ± 780.4
Moderate–severe HIE neonate	1085.5 ± 812.5	94.6 ± 164.1	157.1 ± 568.5	1572.8 ± 3432

### Serum Biomarker Panel Concentrations in Neonates With a Low Cord pH Based on pH, Lactate, Base Deficit, and Sentinel Events

Eighteen neonates with low cord pH, with or without mild HIE, were further analyzed using physiologic parameters such as pH, lactate, base deficit, and the presence of sentinel events to examine if any of these parameters correlated with the concentrations of the four neuroproteins in the biomarker panel. The cohort of 18 neonates was divided into 2 groups for each of these 4 parameters: a pH ≤7 (*n* = 3) compared to a pH of 7 or higher (*n* = 15), a serum lactate concentration of ≥7 (*n* = 5) compared to <7 (*n* = 13), a base deficit equal to or greater than 13 (*n* = 7) compared to <13 (*n* = 11), and neonates with sentinel events (*n* = 3) compared to neonates without sentinel events (*n* = 15). The criteria for grouping of the subjects were chosen because a pH <7 is associated with a higher risk of long-term neurologic deficits, a lactate >7 is associated with a higher risk of encephalopathy, and a base deficit >13 represents a severe metabolic acidosis (22–25). The presence of a sentinel event allowed for some understanding of the timing of the rise in the biomarkers. Sentinel events were defined as umbilical cord mishap (cord prolapse), uterine rupture, placental abruption, shoulder dystocia and major maternal hemorrhage, trauma, cardiorespiratory arrest, or seizures immediately preceding delivery (26).

**Figure 3:**
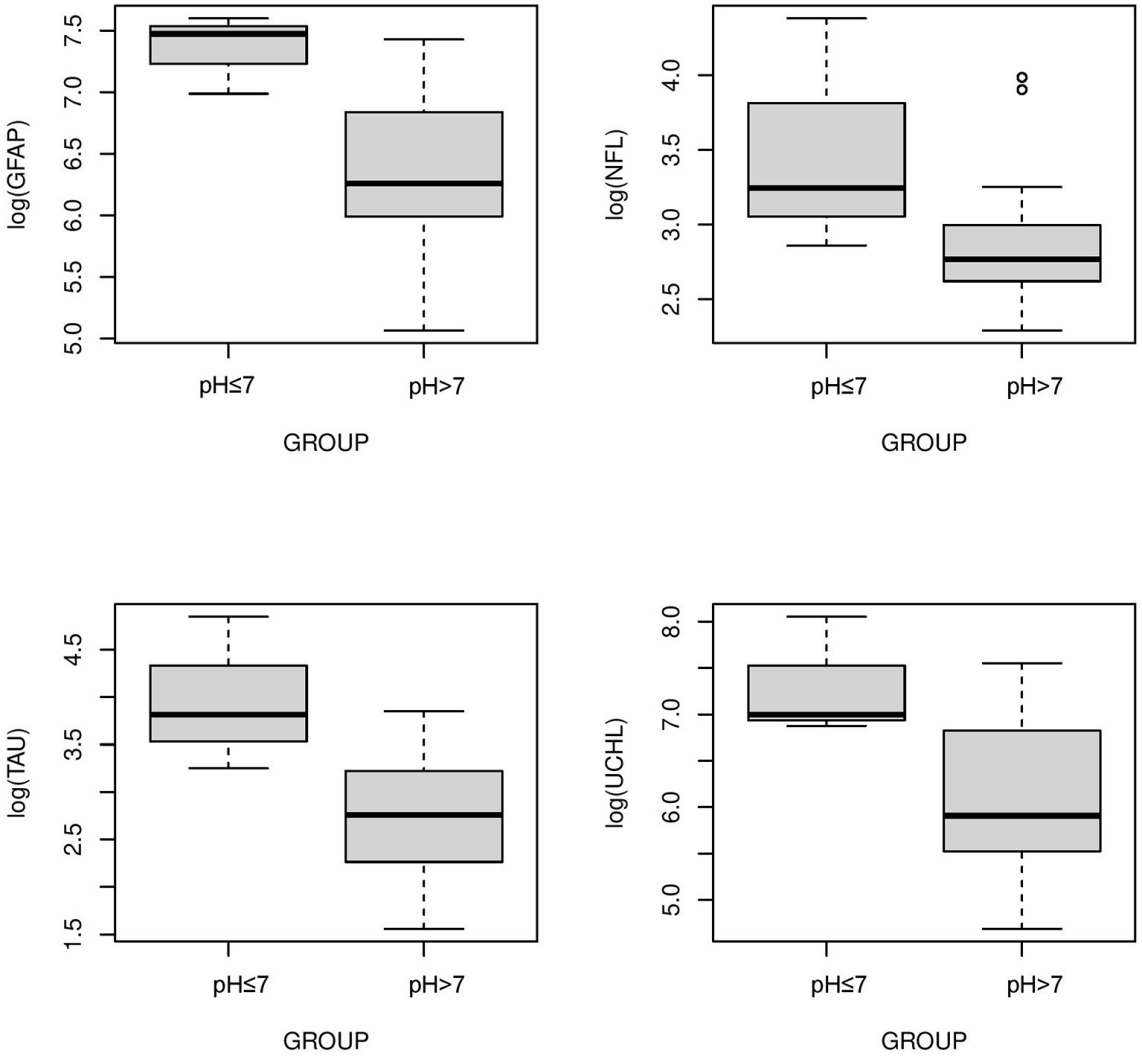
Serum concentrations of GFAP, NFL, Tau, and UCH-L1 in neonates with a *pH* ≤*7 compared to a pH* >*7*. GFAP, Tau and UCHL1 serum concentrations were higher in neonates with a pH ≤7 compared to neonates with a pH >7 (*P* < 0.05) (Mean ± STD). Serum concentration of NFL was similar between neonates with *pH* ≤*7 and neonates with pH* >*7*.

**Figure 4:**
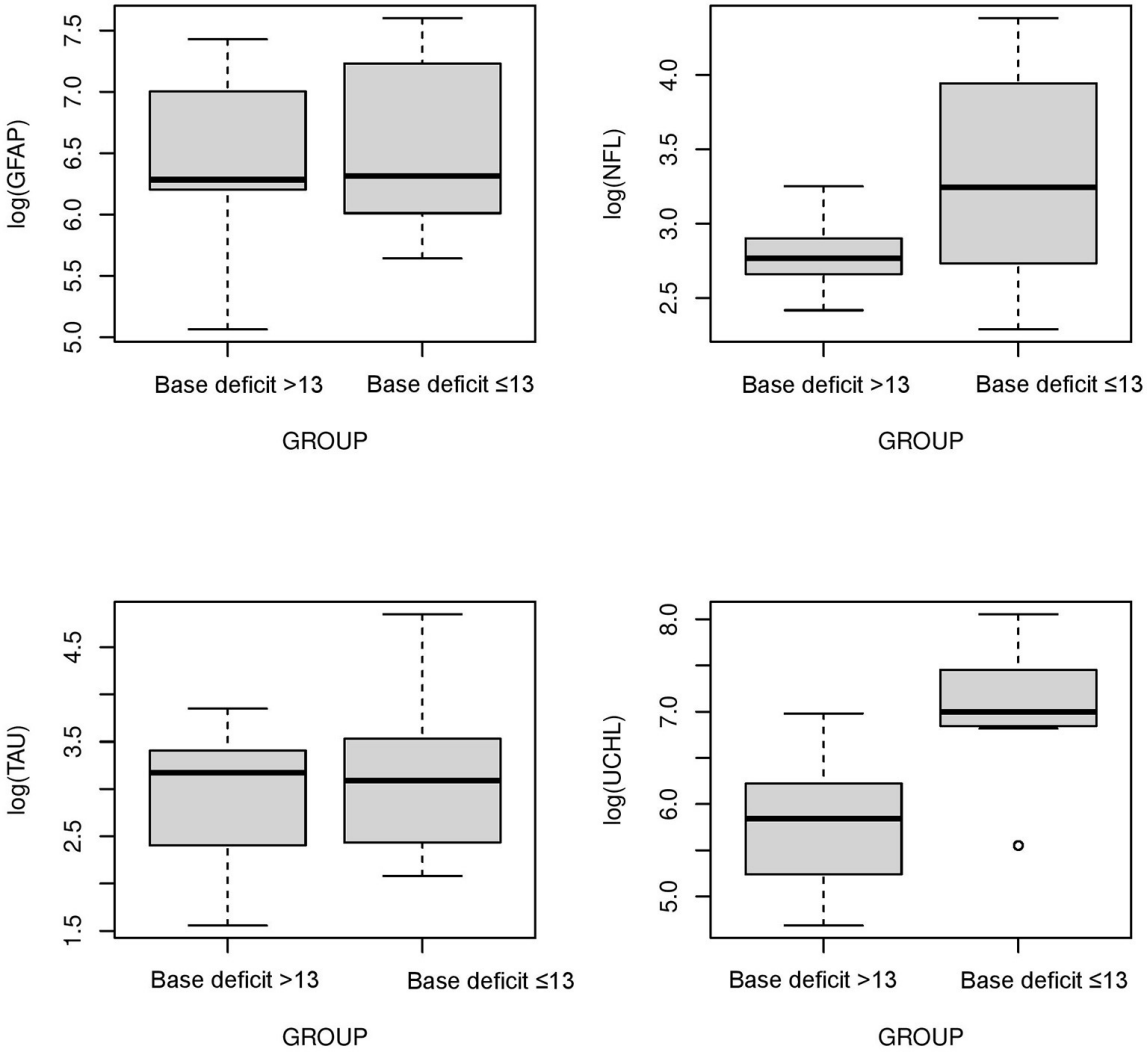
Serum concentrations of GFAP, NFL, Tau, and UCH-L1 in neonates with a base deficit of 13 or greater compared to neonate with base deficit <13. UCH-L1 low concentration was higher in neonates with a base deficit of 13 or greater (*P* < 0.05) (Mean ± STD). Serum concentration of GFAP, NFL and Tau were similar between neonates with base deficit of 13 or greater and base deficit <13.

The serum concentrations of GFAP Tau, and UCH-L1 were higher in neonates with a pH ≤7 compared to neonates with a pH higher than 7 (*P* < 0.05, Figure 3). No difference in serum concentration of NFL was noted in neonates with pH ≤7 compared to neonates with pH higher than 7. UCH-L1 concentration was higher in neonates with a base deficit of 13 or greater (*P* < 0.05, Figure 4). Neonates with a known sentinel event had no difference in serum biomarkers when compared to neonates without history of sentinel events. No differences in biomarker profile were noted in neonates with lactate ≤7 vs. neonates with lactates more than 7.

### Serum Concentrations of a Biomarker Panel in Neonates With a Low Cord pH With Mild HIE Compared to a Low Cord pH Without Mild HIE

The cohort of 18 neonates was divided into two groups, neonates with a normal neurologic exam, Sarnat 0 (*n* = 10, without mild HIE), and neonates with a Sarnat 1 exam (*n* = 8, mild HIE). No difference in biomarker profile was noted between neonates with Sarnat 0 vs. neonates with Sarnat 1.

### Serum Concentrations of miRNAs in Control Neonates, Neonates With Low Cord pH and Neonates With Moderate to Severe HIE

A panel of 68 miRNAs were analyzed among these 3 cohorts from the blood samples obtained at 0–6 h of life. When comparing the neonates with low cord pH neonate group to control neonates, 41 out of 68 miRNA concentrations were different (*P* < 0.05). Of these 41 miRNAs, 36 were upregulated and 5 were downregulated in the low cord pH neonates when compared to the controls. In comparing neonates in the moderate-severe HIE group to control neonates, 43 out of 68 miRNA concentrations were different (*P* < 0.05). Out of these 43 miRNAs, 40 were upregulated and 3 were downregulated in the moderate-severe HIE group compared to the healthy controls (Table 4).

**Table 4:** *p*-value of miRNA concentration in mild HIE verses healthy control, moderate to severe HIE verses healthy control and mild HIE verses moderate-severe HIE respectively.

	**miRNA**	**Concentration of miRNA** **in mild HIE compared** **to control**	**Concentration of mRNA** **in moderate-severe HIE** **compared to control**	**Significance** **of miRNA**
1	hsa.let.7b.5p	↑	↑	Suppress apoptosis and autophagy of mesenchymal stem cells (27)-Human
2	hsa.let.7d.5p	↑	↑	Involved in cell proliferation, invasion, angiogenesis and tumor metastatasis (28)-Human
3	hsa.let.7f.5p	↑	↑	Restores ischemia induced neovascularization (29)-Mouse
4	hsa.let.7g.5p	↑	↑	Inhibits cell migration and cell growth in hepatocellular carcinoma (30)-Human
5	hsa.let.7i.5p	↑	↑	Attenuates human brain microvascular damage (31)-Human
6	ath. mir167d	↑	↑	Studies in plants
7	hsa.mir.103a.3p	↑	↑	Contributes to angiotensin II induced renal inflammation (32)-Human
8	hsa.mir.107	↑	↑	Regulate CDK5R1 expression in post mitotic neurons (33)-Human
9	hsa.mir.124.3p	≈	↓	Reduces caveolar density in porcine kidney (34)-Pig
10	hsa.mir.125b.5p	≈	↑	Regulates IL-1b induced inflammatory gene (35)-Human
11	hsa.mir.128.3p	↑	↑	Elevated in salmonella infection-decrease macrophage recruitment (36)-Human
12	hsa.mir.1285.5p	↓	≈	Unknown
13	hsa.mir.132.3p	↑	↑	Involved in endothelial tube formation and reduce myofibroblast differentiation (37)- Mouse
14	hsa.mir.134.5p	↑	↑	Mediates gene silencing (38)-Human
15	hsa.mir.142.3p	↑	↑	Negative regulation of IL-1 alpha production (39)-Human
16	hsa.mir.145.5p	↑	↑	Positive regulation of cellular response to hypoxia (40)-Human
17	hsa.mir.146a.5p	↑	↑	Negative regulation of IL-6 production (41)-Human
18	hsa.mir.150.5p	↑	↑	Regulation of vascular endothelial cell proliferation (42)-Human
19	hsa.mir.151a.3p	↑	↑	Promotes proliferation, epithelial-to-mesenchymal transition (43)-Human
20	hsa.mir.155.5p	↑	↑	Prevents necrotic cell death in cardiomyocyte progenitor (44)-Human
21	hsa.mir.15a.5p	↑	↑	Impair human circulating proangiogenic cell functions (45)-Human
22	hsa.mir.15b.3p	↑	↑	Enhances tumorigenesis and malignant transformation (46)-Human
23	hsa.mir.15b.5p	↑	↑	Targets amyloid precursor protein- Alzheimer's disease (47)-Human
24	hsa.mir.16.2.3p	↑	↑	Suppresses RAR-β (2) expression, increases tumor cell proliferation (48)-Human
25	hsa.mir.16.5p	↑	↑	regulate p53 signaling pathway (49)-Human
26	hsa.mir.17.5p	↑	↑	Suppresses toll-like receptor signaling in human leukemia cells (50)-Human
27	hsa.mir.181b.5p	↑	↑	Regulates ALX/FPR2 receptor expression in macrophages (51)-Human
28	cel.mir.70.3p	↓	↓	Unknown
29	hsa.mir.191.5p	↑	↑	Regulators of brain-derived neurotrophic factor (52)-Human
30	hsa.mir.195.5p	↓	↑	Impairs insulin signaling and glycogen metabolism (53)-Human
31	hsa.mir.197.3p	↑	↑	Negative regulation of interleukin-18 production (54)-Human
32	hsa.mir.206	↑	↑	Silences the expression of Connexin 43(55)-Human
33	hsa.mir.20a.5p	↑	↑	Suppresses IL-17 production (56)-Human
34	hsa.mir.210.3p	↑	↑	Upregulates neuronal pentraxin 1 post hypoxic event (57)-Human
35	hsa.mir.214.3p	↑	↑	Suppresses XBP1-Mediated Endothelial Cells ANGIOGENESIS (58)-Human
36	hsa.mir.21.5p	↑	↑	Angiogenesis in Diabetic Retinopathy via PPARα (59)-Human
37	hsa.mir.22.3p	↑	↑	Induces endothelial progenitor cell senescence (60)-Human
38	hsa.mir.23a.3p	↑	↑	Apoptosis of cerebral vascular endothelial cells by down-regulating ZO-1 (61)-Human
39	hsa.mir.24.3p	↑	↑	Negative regulation of interferon-gamma production (62)-Human
40	hsa.mir.26b.5p	↑	↑	Negative regulation of chemokine production (63)-Human
41	hsa.mir.29b.3p	↑	↑	Negative regulation of cytokine-mediated signaling pathway (64)-Human
42	hsa.mir.301a.3p	↑	↑	Regulation of vascular associated smooth muscle cell proliferation (65)-Human
43	hsa.mir.30e.5p	↑	↑	Negative regulation of cardiac muscle cell apoptotic process (66)-Human
44	hsa.mir.323a.3p	↑	↑	Negative regulation of amyloid precursor protein (67)-Human
45	hsa.mir.328.3p	↑	↑	Contributes to adverse electrical remodeling in atrial fibrillation (68)-Human
46	hsa.mir.331.5p	↓	↓	Regulates Cell Proliferation and Glucose Metabolism (69)-Human
47	hsa.mir.338.3p	↑	↑	Negative regulation of cell migration (70)-Human
48	hsa.mir.342.3p	↑	↑	Unknown
49	hsa.mir.346	↓	↓	Regulation of amyloid precursor protein (71)-Human
50	hsa.mir.34a.5p	↑	↑	Regulating steatosis by targeting PPARα expression (72)-Human
51	hsa.mir.34b.3p	↓	↓	Regulation of transporter activity (73)-Human
52	hsa.mir.34c.5p	↓	≈	Epithelial-mesenchymal transition in endometriosis (74)-Human
53	hsa.mir.370.3p	↑	↓	Suppresses the expression and induction of CYP2D6 (75)-Human
54	hsa.mir.382.5p	↑	↑	Unknown
55	hsa.mir.451a	↑	↓	Negative regulation of transporter activity (76)-Human
56	hsa.mir.483.3p	≈	↑	Simulate the probiotic effect of E. coli Nissle 1917 on T84 epithelial cells (77)-Human
57	hsa.mir.486.5p	↓	↑	Suppresses Cell Growth With the Involvement of a Target PIK3R1 (78)-Human
58	hsa.mir.491.5p	↓	↓	Inhibit cellular invasion in U251 and U87 glioma cells (79)-Human
59	hsa.mir.497.5p	↑	↑	Targets the TNF-α/NF-κB pathway (80)-Human
60	hsa.mir.5010.3p	↓	↓	Unknown
61	hsa.mir.532.5p	↑	↑	Unknown
62	hsa.mir.545.3p	≈	≈	Unknown
63	hsa.mir.7.5p	↑	↑	Negative regulation of amyloid-beta clearance (81)-Human
64	hsa.mir.7417.5p	≈	↑	Unknown
65	hsa.mir.885.5p	≈	↑	Suppressing the expression of lipid receptor and sterol transporter (82)-Human
66	hsa.mir.92a.1.5p	↓	↓	Decreases angiogenesis (83)-Human
67	hsa.mir.93.5p	↑	↑	Negative regulation of cell population proliferation (84)-Human
68	hsa.mir.98.5p	≈	↑	Modulates Cytokine Production in Systemic Lupus Erythematosus by Targeting IL-6 (85)-Human

## Discussion

This study evaluated the serum concentrations of a novel panel of four neuroprotein biomarkers and 68 miRNAs from neonates with low cord pH, with and without evidence of mild HIE, compared to healthy control neonates and neonates with moderate to severe HIE. The serum concentrations of biomarkers GFAP, NFL and Tau were increased in the low cord pH group and the moderate to severe HIE group compared to the control group at 0–6 h of life. The serum concentrations of biomarkers UCH-L1 were higher in neonates with moderate to severe HIE compared to the control group. When physiologic parameters were examined in neonates with low cord pH, serum concentration of GFAP, Tau and UCH-L 1 were higher in neonates with a pH ≤7 compared to neonates with pH more than 7. UCH-L1 was higher in neonates with a base deficit of 13 or greater compared to neonates with base deficit <13. No difference in biomarker profile was noted in neonates with or without sentinel events and neonates with lactate greater or equal to 7 verses neonates with lactate <7. Often the exact timing of the potential neurologic injury was unknown. This lack of injury timing adds variability to the interpretation of biomarkers' serum concentrations following birth. To the best of our knowledge, this prospective study is the first to examine a brain-specific screening method to help clinicians identify neonates with the potential for brain injury using cord pH and a panel of serum neuroproteins. In addition, this study's comparison of a panel of 68 miRNA serum concentrations between 3 cohorts is the most extensive to date.

Chalak et al. compared the umbilical cord concentrations of biomarkers like GFAP and UCHL-1 in 7 neonates with mild HIE with 20 neonates with moderate-severe HIE. They found that concentration of GFAP and UCH-L1 increased with severity of HIE (86). Our study had similar finding. NFL concentration was higher in neonates with moderate-severe HIE compared to neonates with low cord pH/mild HIE. Unlike the Chalak study, our study included a control group of healthy neonates. Our study found increased serum concentrations of NFL in both cohorts of mild and moderate-severe HIE compared to healthy controls at 0–6 h of life.

Shah et al. studied the temporal change in NFL concentrations in neonates with mild, moderate, and severe HIE (17). The study recruited 11 term neonates with acidosis and/or mild HIE and neonates with moderate to severe HIE who met the criteria for therapeutic hypothermia. Of the neonates with mild HIE, 3 out of 11 babies had a detectable or raised NFL level at 24 h of life (17, 87). Our study had 18 neonates with acidosis, with or without mild HIE. NFL serum concentrations were higher in neonates with mild HIE compared with controls but lower than neonates with moderate to severe HIE who qualified for therapeutic hypothermia. Our data demonstrated an elevation in NFL earlier than 24 h. This finding suggests that during the 6 h that an acidotic neonate, with or without mild HIE, is undergoing evaluation for a neuroprotective intervention, NFL may be useful to distinguish neonates with injury.

In our study, neonates with moderate-severe HIE had increased serum concentrations of GFAP, NFL UCHL-1, and Tau compared to healthy controls. This study, our prior studies, and the works of others demonstrate that neonates with moderate-severe HIE undergoing hypothermia have increased concentrations of these four biomarkers compared to control subjects (86, 88, 89).

The cohort of neonates with a low cord pH were divided based on physiologic parameters including pH, lactate, and base excess. These physiologic parameters have been associated with outcomes and were used because we lacked short-term MRI data or long-term developmental testing (22–25). Three out of four serum biomarkers were higher in neonates with a pH ≤ 7. We also compared neonates with known sentinel events to neonates who did not have a sentinel event In neonatal HIE biomarker research, the exact timing of injury is often unknown (90, 91). Biomarker serum concentrations change over time. The lack of exact timing may cause dilution of the concentration when looking at a cohort because those neonates with sentinel events may be earlier in the injury pathophysiology compared to those neonates without a sentinel event. We did not find differences in biomarkers in neonates with or without sentinel events. Of note, we had only 3 neonates with sentinel events vs. 15 neonates without sentinel events. Our previous research showed that UCH-L1 peaks within 0–6 h (12).

In animal models of hypoxia-ischemia, miRNA 210 has a role in regulating microglia-mediated neuroinflammation (92). Based on a neonatal rat model, Bo Li et al. studied the pathway of miRNA following hypoxia-ischemia and identified miR-210 as a novel regulator of microglial activation. This study demonstrated that miR-210 expression is induced in activated microglia after hypoxic ischemia. This miR-210 induction leads to an enhanced innate proinflammatory immune response (21). Other miRNAs have been studied in adult stroke, neurogenic disorders, and cancer pathogenesis. Studies have demonstrated involvement of miRNAs such as miR-150, miT-181a, miR-17-92 T cell and B cell differentiation (93). After recognition of toll-like receptors, miRNAs such as miR-146, miR-155, and miR-223 activate innate and acquired immune responses (94). A study by Sullivan et al. showed that neonates with HIE had decreased concentrations of 3 miRNAs, namely miR-374a-5p, miR-376c-3p, miR-181b-5p, when compared to healthy controls (20). Looney et al. also demonstrated downregulation of miR-181b in neonates with HIE compared to controls (95). In our study, when compared to controls, the concentration of miR-181b-5p was higher in neonates with mild HIE (*P* < 0.01) and moderate-severe HIE (*P* < 0.001). In addition, when compared to controls, concentrations of MiR-210 were higher in mild HIE (*P* < 0.02) and moderate-severe HIE (*P* < 0.001). Casey et al. studied miRNA expression in a piglet model of HI and demonstrated 32 out of 55 miRNAs were upregulated and 23 out of 55 were downregulated. However, this study was unable to show differences in miRNA between mild HIE vs. moderate and severe HIE (96). Our study demonstrated 36 out of 68 miRNAs were upregulated and 5 out of 68 were downregulated in neonates with mild HIE compared to healthy controls. In addition, we detected 40 out of 68 miRNAs upregulated and 3 out of 68 miRNAs downregulated in neonates with moderate-severe HIE compared to healthy controls. Our study clearly showed that the concentrations of miRNAs are different in healthy controls vs. neonates with mild HIE and moderate-severe HIE. Expression of miRNA could be an on/off phenomenon after hypoxia-ischemia. Therefore, miRNAs can be useful markers to differentiate neonates with any severity of HIE from healthy control neonates.

As outlined above, few published studies exist that have examined prospective biomarker concentrations in neonates with metabolic acidosis at birth with or without mild HIE. Most of the biomarker studies have measured the concentrations of biomarkers in neonates with moderate-severe HIE undergoing therapeutic hypothermia. Some of these studies included only a small number of neonates with mild HIE who did not qualify for hypothermia and compared them with neonates with moderate-severe HIE rather than control subjects. This study is the first to explore the serum concentrations of a panel of 4 biomarker profiles and to compare these concentrations in neonates with mild HIE, healthy controls, and neonates with moderate-severe HIE. This study is also the first to examine a panel of 68 miRNAs. Neonates with mild HIE have subtly abnormal neurologic exams with borderline lab parameters that give a false impression of unklikely brain injury, even though multiple prior studies have shown that 25% of these neonates might have abnormal neurologic outcomes. As our data demonstrates, a panel of biomarkers with miRNAs could be a useful tool for clinicians to identify neonates with mild HIE with evidence of some form of brain injury. Our samples were collected within 0–6 h and preferably within the first hour of birth. The early differentiation of this sub-cohort of neonates with mild HIE that have higher concentrations of GFAP, NFL and Tau might be a useful tool to identify neonates with brain injury. These identified neonates could benefit from interventions such as therapeutic hypothermia or erythropoietin therapy. However, further studies must establish these biomarkers' serum concentrations that can best predict brain injury with increased sensitivity and specificity.

Our study had limitations. The number of neonates with a low cord pH was smaller compared to neonates in the moderate-severe HIE and healthy control cohorts. In addition, the neonates with low cord pH did not undergo an MRI or developmental testing as part of their care. To effectively use the panel of biomarkers' serum concentrations for bedside decision making, a short-term outcome of brain injury identified on MRI and a long-term outcome measured at 22–24 months with a Bayley score are required. Despite the limitations, this pilot study yielded important data that show a panel of biomarkers offers promise in assisting the bedside clinician in selectively identifying neonates with brain injury.

## Conclusion

A biomarker panel approach in neonates with a low cord pH may help the bedside clinician with real-time decision making. However, this observation warrants further evaluation in a larger cohort to examine the relationship between serum biomarker concentrations and outcomes both short- (MRI injury) and long-term (Bayley scores at 22–24 months of age).

## Data Availability Statement

The raw data supporting the conclusions of this article will be made available by the authors, without undue reservation.

## Ethics Statement

The studies involving human participants were reviewed and approved by University of Florida. Written informed consent to participate in this study was provided by the participants' legal guardian/next of kin.

## Author Contributions

PG and ZY were responsible for project design, data cleaning, data analysis and interpretation, and drafting of the article. LS was responsible for data acquisition and manuscript editing. NB and ZY were responsible for data analysis and graphing. CR was responsible for tissue processing, storage, and handling. MW was responsible for concept of the project, data analysis and interpretation, drafting the article, and final approval of the version to be published. All authors contributed to the article and approved the submitted version.

## Funding

This study was funded by the University of Florida.

## Conflict of Interest

The authors declare that the research was conducted in the absence of any commercial or financial relationships that could be construed as a potential conflict of interest.

## Publisher's Note

All claims expressed in this article are solely those of the authors and do not necessarily represent those of their affiliated organizations, or those of the publisher, the editors and the reviewers. Any product that may be evaluated in this article, or claim that may be made by its manufacturer, is not guaranteed or endorsed by the publisher.

## Abbreviations

HIE: Hypoxic-Ischemic Encephalopathy
UCH-L1: Ubiquitin carboxyl-terminal esterase L1
GFAP: Glial fibrillary acidic protein
NFL: Neurofilament light chain
miRNAs: MicroRNAs
NICU: Neonatal Intensive Care Unit.

## Definitions

**Low cord pH neonates:** Neonates with cord pH (arterial/venous) ≤7.1 or neonates with cord pH (arterial/venous) between 7.11–7.15 with worsening sarnat exam during serial assessment in the first 6 h of life with evidence of mild hypoxic ischemic encephalopathy.

**Perinatal adverse events:** Events occurring during perinatal period with potential to result in acute disruption of placental blood flow such as uterine rupture, placental abruption, umbilical cord prolapse, shoulder dystocia, maternal cardiac arrest, amniotic air embolism etc.

**Mild hypoxic ischemic encephalopathy:** Neonates with low cord pH with one or more abnormal category in modified sarnat staging criteria within 6 h of life and does not have evidence of moderate-severe encephalopathy.

**Moderate-severe hypoxic ischemic encephalopathy:** Neonates with cord pH <7 and/or a base deficit >16 with evidence of moderate-severe encephalopathy (defined as seizures or 3 or more abnormalities on a modified sarnat exam), or pH between 7.01–7.15 and/or base deficit between 10–15.9 and acute perinatal adverse event and an APGAR of 5 or less at 5 min or mechanical ventilation at 10 min of life if no blood gas was available.
